# Skeletal Muscle Signaling Following Whole-Body and Localized Heat Exposure in Humans

**DOI:** 10.3389/fphys.2020.00839

**Published:** 2020-07-14

**Authors:** Mohammed Ihsan, Louise Deldicque, John Molphy, Florian Britto, Anissa Cherif, Sebastien Racinais

**Affiliations:** ^1^Research and Scientific Support, Aspetar Orthopaedic and Sports Medicine Hospital, Doha, Qatar; ^2^Institute of Neuroscience, Université catholique de Louvain, Louvain-la-Neuve, Belgium

**Keywords:** muscle mass, heat treatment, hypertrophy, mitochondrial biogenesis, heat shock protein, muscle atrophy

## Abstract

This study identified the changes in hypertrophy/atrophy and mitochondrial-related signaling in human skeletal muscle following whole-body (WB) and localized single leg (SL) heat treatment. Nine active male participants were administered either 60 min of passive WB (44–50°C, 50% humidity) or SL (water-perfused suit at 49.5 ± 1.4°C) heat treatment at least 1 week apart in a counterbalanced order. The untreated leg during SL was considered as control (CON). Core, skin, and quadriceps muscle temperature were monitored throughout the experimental trials. Muscle microbiopsy samples were obtained prior to (PRE), and 30 min and 3 h post (POST) following heat treatment. Muscle temperature increased with time (*p* < 0.0001) in both WB and SL, with no differences between conditions (38.8 ± 0.5°C vs. 38.1 ± 0.6°C, *p* = 0.065). Core temperature increased only following WB, and was significantly higher compared with SL (39.1 ± 0.3°C vs. 37.1 ± 0.1, *p* < 0.0001). Compared with PRE, WB up-regulated the phosphorylation status of the majority of the Akt/mTOR pathway (Akt, mTOR, S6K1, rpS6, and p-eIF4E; *p* ≤ 0.050), with the exception of 4EBP1 (*p* = 0.139). WB also increased the mRNA of HSPs 72, 90, and 25 (all *p* < 0.021), and increased or tended to increase the phosphorylation of FOXO1 (*p* = 0.066) and FOXO3a (*p* = 0.038). In addition, most (NRF1, NRF2, COX2, and COX4-I2; all *p* ≤ 0.050), but not all (CS, Cyt c, and COX4-I1; *p* > 0.441) mRNA content indicative of mitochondrial biogenesis were increased following WB, with no changes evident in these parameters in SL or CON (all *p* > 0.090). These results indicate that 1 h of WB heat treatment enhanced anabolic (Akt/mTOR), mitochondrial, and cyto-protective signaling (HSP), with a concomitant possible inhibition of FOXO transcription factors.

## Introduction

While resistance exercise/loading is the most notable method to initiate muscle hypertrophy (Francaux and Deldicque, 2019), there has been substantial interest in developing non-exercise modalities to increase/preserve muscle mass in various clinical populations. Within such research, the use of passive heat stress has emerged as a promising adjunct therapy, where within rodent and cell culture models, has been shown to attenuate the loss in muscle mass in aging (Ohno et al., 2012), disuse (Selsby and Dodd, 2005), and pharmacological (Tsuchida et al., 2017) models of muscle atrophy. The potential mechanisms underpinning such adaptations are likely triggered by an increased expression of heat shock proteins (HSP) (Goto et al., 2003; Ohno et al., 2015; Obi et al., 2019), subsequently acting on a myriad of signaling pathways including anabolic signaling through the protein kinase B (Akt)/mammalian target of rapamycin (mTOR) pathway (Yoshihara et al., 2013), attenuated catabolic signaling through the forkhead box (FOXO) transcription factors (Yoshihara et al., 2016), and mitochondrial-related signaling, possibly involving the transcriptional coactivator peroxisome proliferator-activated receptor gamma coactivator-1α (PGC-1α; Ihsan et al., 2014; Tamura et al., 2014; Cannavino et al., 2015).

Recent studies indicate that heat stress may confer similar benefits in humans, although research exploring the influence of treatment modality (i.e., localized vs. whole body heat exposure), and the mechanisms involved are rather at their infancy. For instance, short-term (2 h/day for 6–10 days) localized heat therapy targeting the quadriceps has been shown to attenuated decline in muscle mass in participants immobilized over a 2-week period (Hafen et al., 2019), with longer term (8–10 weeks) treatment resulting in increased muscle mass, muscle capillarity, and improved knee extensor muscle strength in healthy weight-bearing participants (Goto et al., 2011; Kim et al., 2020). While these studies support the therapeutic potential of localized heat treatment, the mechanisms involved show discord, with some showing evidence of a HSP-mitochondrial-centered mechanism (Hafen et al., 2018, 2019), whilst others demonstrating limited involvement of such pathways in human models of localized heat therapy (Kim et al., 2020). Similar discrepancies are observed in passive whole-body (WB) modalities, where acute heat stress has been shown to repress PGC-1α mRNA, a key regulator of mitochondrial adaptations (Petrie et al., 2016). Yet, longer term WB heat treatment (11 days to 6 weeks, 40–60 min/day) has been shown to improve aerobic capacity, muscle capillarity, as well as increase maximal voluntary torque, despite unchanged voluntary activation (Racinais et al., 2017; Hesketh et al., 2019). This indicates that multiple mechanisms (e.g., Akt/mTOR, FOXO) may be involved in regulating the improved oxidative and contractile function following localized and WB treatment.

Identifying the signaling response following WB and localized heat treatment in humans enables the identification of the early regulatory mechanisms, and lends understanding to the divergent mechanistic findings previously reported following localized and WB heat therapy. Such information is fundamental to understand the therapeutic potential of such modalities, as well as transfer the findings from rodents and cell culture studies into an applicable adjunct therapy for both sport and clinical situations (e.g., injury, illness, and immobilization). The purpose of this study is therefore to identify the changes in Akt/mTOR signaling, FOXO mediated signaling, HSP regulation, and mitochondrial-related signaling in human skeletal muscle following WB and localized heat treatment.

## Materials and Methods

### Participants

Nine recreationally active male participants (mean ± SD; age: 35 ± 4 years, height: 177 ± 7 cm, mass: 78.9 ± 7.5 kg) completed this study. They were active in endurance or team sports (i.e., 2–4 h training per week) but not involved in any structured training program. Participants were not using any medication and had no lower limb musculoskeletal injuries for at least 12 months prior to the study. They were asked to refrain from all exercise, as well as alcohol and caffeine for at least 24 h prior to the experimental sessions. Participants satisfied the Medical History Questionnaire and Physical Activity Readiness Questionnaire (PAR-Q) before being admitted to the study. They were fully informed of the requirements and risks associated with the study, and a written informed consent was obtained before participation. This study was approved by the Anti-Doping Laboratory Qatar research ethics committee (approval no. F20170000252).

### Experimental Design

**FIGURE 1:**
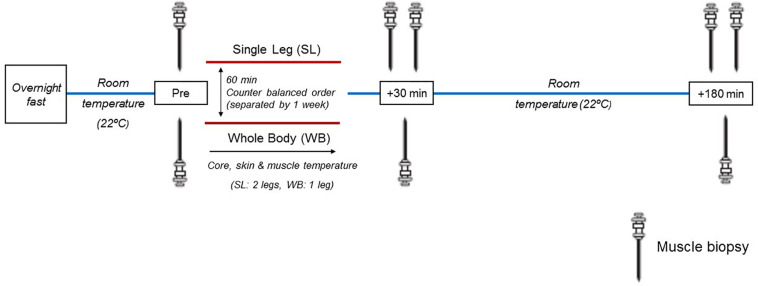
Schematic representation of the experimental design. Muscle biopsies were sampled before heat treatment (Pre), and at generic time-points of 30 and 180 min following heat treatment. During SL, additional biopsies were obtained from the non-heated leg at 30 and 180 min post.

All participants underwent two separate experimental sessions 1 week apart, to ensure adequate healing from the muscle biopsies and muscle temperature measures. Upon arrival, participants were instrumented with rectal and skin thermistors measuring core (T_re_) and skin temperatures (T_s_), respectively. In addition, their vastus lateralis muscle was prepared for the measurement of intramuscular temperature (T_m_) and the extraction of muscle biopsy samples. Participants were then administered 60 min of either WB or localized single-leg (SL) heat exposure, in a counterbalanced order (Figure 1). Following treatment, participants rested in a temperate laboratory (21.7 ± 1.8°C, 58 ± 6% relative humidity) for a further 3 h. All temperature measurements were monitored throughout heat exposure and recovery. Muscle biopsies were obtained prior to (PRE) both treatments as described below. Due to the uncertainty of the effect of heat stress on the time-course response of the protein and genes of interest in the current study, and given the temporal differences between the different pathways investigated, we obtained additional biopsy samples at generic time-points of 30 min and 3 h following both treatments, and undertook our analysis on the peak response observed within this time frame (POST). This approach minimized the inter-individual response and temporal variability associated with investigating multiple pathways, and enabled us to assess distinctively the regulation of these pathways in response to WB or SL. Regardless, representative western blot images for all time points are included in the Supplementary Content. The biopsies were obtained from the same leg during WB and SL, counterbalanced between participants according to their dominant and non-dominant leg. To account for the possible effect of time *per se* on changes in gene expression and the phosphorylation status of the proteins, additional biopsies were also obtained from the contralateral untreated leg (CON) at the POST time-points during SL. We refrained from taking a pre-biopsy from CON during SL to limit the stress experienced by our participants. Overall, a total of eight biopsies were obtained (five during SL and three during WB). All experimental sessions were conducted at the same time of the day (∼0800), following an overnight fast lasting 12 h. Participants recorded their last calorie intake from the evening prior to reporting to the laboratory, and replicated it prior to second trial. A visual inspection of the participants’ logs was undertaken upon their arrival for the second trial for confirmation, and no participants were excluded from the study on this basis. Participants consumed 300–500 ml of water 2 h prior to their arrival at the laboratory.

#### Heat Treatment

Whole body heat treatment was undertaken according to previous methods (Racinais et al., 2017), where participants were housed in an environmental chamber (Sanwood, China) initially set at 50°C and 50% RH. If reaching a T_re_ of 39.0°C, the environmental temperature was adjusted between 44 and 50°C to maintain a T_re_ clamp at approximately 39°C for the remainder of the duration. Changes in T_m_ are expected to approximate changes in T_re_ using this passive heating model, resulting in modest differences between T_m_ and T_re_ (Racinais et al., 2017). During SL, participants remained in standard laboratory environment (∼22°C, 60% relative humidity) but were instrumented with a customized water-circulating sleeve, designed to cover the calves and thighs up to the level of the gluteal fold. The sleeve is made of a tight-fitting fabric, incorporated with an extensive network of polyvinyl chloride tubing. The sleeve was connected to a bath circulator maintained at 49.5 ± 1.4°C, and perfused through the garment for 60 min. The mean tubing temperature during SL was 45.3 ± 2.2°C. Fluid intake was allowed at libitum (temperate water) throughout both conditions.

#### Core, Skin, and Muscle Temperature

Core temperature was measured using a sterile temperature probe (MRB rectal probe, Ellab, Hillerød, Denmark), self-inserted 12 cm past the anal sphincter, and connected to a precision digital thermometer measuring to the nearest 0.1°C (DM 852, Ellab A/S, Hillerød, Denmark). Skin temperatures were determined using iButton temperature sensors/data loggers (iButtonTM, Maxim Integrated Products, Sunnyvale, CA, United States), secured to the participants’ chest, arm, thigh, and calf using non-porous adhesive tape. Mean skin temperature was determined using the following formula (Ramanathan, 1964): T_s_ = (0.3 × T_chest_) + (0.3 × T_biceps_) + (0.2 × T_thigh_) + (0.2 × T_calf_). Thigh and calf skin temperatures were sampled on both the heated and non-heated leg during SL, and the average of both values were used to determine the mean T_s_. Muscle temperature (T_m_) was continuously measured using an indwelling flexible thermistor (MAC flexible probe, Ellab, Hilleroed, Denmark), inserted into muscle belly of the vastus lateralis via a 16-gauge catheter under topical anesthesia (5% lidocaine). The T_m_ measurements were sampled at a ∼3 cm depth, approximately 17 cm proximal to the patella border, along the greater trochanter-patella border axis.

#### Muscle Biopsies

Muscle samples were extracted using a disposable, spring-loaded microbiopsy system (MAX-CORE, Bard Biopsy Systems). After the application of topical anesthesia (5% lidocaine) around the sampling region, a 13-gauge cannula was inserted to a 3 cm depth, approximately 2–3 cm distal to the measurement site of T_m_. A 14-gauge biopsy needle was then inserted into the cannula, and three muscle samples, totaling approximately 20–30 mg were subsequently extracted per biopsy. The tissue samples were immediately frozen in liquid nitrogen and stored in a −80°C freezer for later analysis.

#### Tissue Processing and Western Blotting

Approximately 20–25 mg of muscle samples was homogenized with a Polytron mixer in an ice-cold lysis buffer [20 mM Tris–HCl (pH 7.0), 270 mM sucrose, 5 mM EGTA, 1 mM EDTA, 1 mM sodium orthovanadate, 50 mM beta-glycerophosphate, 5 mM sodium pyrophosphate, 50 mM sodium fluoride, 1 mM DTT, 1% Triton X-100, and a complete protease inhibitor tablet], centrifuged at 10,000 *g* for 10 min at 4°C. Then supernatant was portioned into aliquots and stored at −80°C. Total protein content of the muscle homogenate was determined using a commercially available protein assay kit, as per the manufacturer’s instructions. Aliquots were diluted and suspended in a sample buffer and subjected to protein separation via electrophoresis. Following separation, proteins were transferred to a PVDF membrane, prior to membrane blocking in 5% BSA/Tris-buffered saline/0.1% Tween 20 (TBST) for 2 h at room temperature. Membranes were then incubated overnight at 4°C with gentle agitation in blocking solution containing primary antibodies (dilution 1:500 or 1:1000, all from Cell Signaling Technology, Denvers, MA, United States) specific to phosphorylated and total forms; Akt Ser^473^ (#4060) total Akt (#9272), mTOR Ser^2448^ (#2971), total mTOR Ser^2448^ (#2971), S6K1 Thr^389^ (#9234), total S6K1 (#2708), rpS6 Ser^235/236^ (#4856), total rpS6 (#2317), 4EBP1 Thr^37/46^ (#2855), total 4EBP1 (#9644) eIF4F Ser^209^ (#9741), total eIF4E (#9742), NF-κB Ser^536^ (#3033), total NF-κB (#8242), IκBα: NF-κB inhibitor protein alpha (#9242), FOXO1 Thr^24^/FOXO3a Thr^32^ (#9464), FOXO3a Thr^318/321^ (#9465), total FOXO1 (#2880), total FOXO3a (#2497), AMPK Thr^172^ (#2535), ACC Ser^79^ (#11818), total ACC (#3676), p38 MAPK Thr^180^/Tyr^182^ (#9211), and total p38 (#9212). Following overnight incubation, the membranes were washed and incubated with secondary anti-mouse (1:5000) anti-rabbit (1:1000) antibodies for 1 h at room temperature. Following washing, chemiluminescent solution was applied to each blot and optical density measurements was obtained using a phosphoimager and subsequently analyzed using an image analysis software. All phosphorylated and total densitometric values were normalized to ponceau dye. As changes in some, but not all total protein forms were evident following heat treatment, all phosphorylated and total forms are presented separately to maintain consistent reporting of the western blot data throughout the manuscript.

#### Real-Time Polymerase Chain Reaction Analysis

Total RNA was extracted from approximately 15 mg of frozen muscle using a TRI reagent according to the manufacturer’s specification. RNA isolation was achieved according to the manufacturer’s instructions. RNA quality (ratio 260/280 around 1.8) and quantity were assessed by Nanodrop (Thermo Fisher Scientific) spectrophotometry. One microgram of total RNA was reverse-transcribed into complementary DNA (cDNA) using the iScriptcDNA Synthesis Kit (Bio-Rad Laboratories) according to the manufacturer’s protocol. The primer sequences are included in Table 1. Each primer pair was tested for its efficiency, which was found to be between 1.86 and 2.13. PCR analyses were conducted using the following conditions: 2 min at 95°C, followed by 40 cycles of 5 s at 95°C (denaturation) and 30 s at 60°C (annealing/extension). All samples were run in duplicate with an internal standard on each plate to correct for interplate variability. Each reaction was processed in a 10-μl volume containing 4.8 μl SsoAdvanced Universal SYBR Green SuperMix (Bio-Rad, Hercules, CA, United States), 0.1 μl of each primer (100 nM final) and 5 μl cDNA at the appropriate dilution. Melting curves were systematically performed for quality control. As no housekeeping gene was totally stable across all experimental conditions, all results were reported to the quantity of cDNA (Quant-iT^TM^ OliGreen ssDNA Reagent and Kit, Life Technologies) after the reverse transcription for each sample according to the manufacturer’s protocol and as described in Lundby et al. (2005).

**TABLE 1:** Primer sequences.

	**Forward**	**Reverse**
Atrogin-1	GTGGTACTGAAAGTCCTTGAA	CTCTTTGGACCAGTGTACATAA
COX II	CAGACGCTCAGGAAATAGAAA	CGTTGACCTCGTCTGTTATG
COX IV-l1	GAGAGCTTTGCTGAGATGAA	CCGTACACATAGTGCTTCTG
COX IV-I2	GGTCTACGTATTTCCTCCAAAG	CTTCCACTGCTTCTTCTCATAG
CS	CCAACAGAGGAACAGGTATC	GGTGTAGATTGGTGGGAAAG
Cyt c	CCAAATCTCCATGGTCTCTTT	CCCAGATGATGCCTTTGTT
HSP 25	CCTGGATGTCAACCACTTC	GGGCAGCGTGTATTTCC
HSP 72	GGTGCTGACCAAGATGAAG	CTGCGAGTCGTTGAAGTAG
HSP 90	ATCAAACTTGGTCTGGGTATT	GATGTGTCGTCATCTCCTTC
Murf-1	CTATCTGCCTGGAGATGTTTAC	TTTGCAGCCTGGAAGATG
NRF1	GCAAGCTATTGTCCTCTGTATC	GTACTTACGCACCACATTCTC
NRF2	AAACTTCTGTTGCTCAGGTAG	TAAGACACTGTAACTCAGGAATG
PGC-1α	CACTTACAAGCCAAACCAACAACT	CAATAGTCTTGTTCTCAAATGGGGA
PPARα	GCTGCAAGGGCTTCTTT	CTGGCATTTGTTTCTGTTCTTT
VEGF	TTTCTGCTGTCTTGGGTGCATTGG	ACCACTTCGTGATGATTCTGCCCT

### Statistics

Data distribution was assessed using the Shapiro–Wilk test. All temperature measures (i.e., T_m_, T_re_, and T_s_) demonstrated a normal distribution, and were analyzed using a two-way repeated measures ANOVA (i.e., time × condition) with Bonferroni *post hoc* correction. The majority of the protein and mRNA data did not conform to a normal distribution. As such, changes in all protein (phosphorylated and total) and mRNA expression following WB, SL, and CON were analyzed in densitometry units using the Wilcoxon signed rank test (i.e., time effect in each condition). In addition, effect sizes (ES = mean difference/pooled SD) were computed between Pre and Post measures to determine the magnitude of change, with ES > 0.2, ES > 0.5 and ES > 0.8 interpreted as small, medium, and large effects, respectively. Pre biopsy taken during SL was used as the pre value for statistical analysis in CON. Statistical significance was accepted at *p* ≤ 0.05. All analyses were undertaken using SPSS version 22 (IBM SPSS, Chicago, IL, United States) or an excel spreadsheet (for ES).

## Results

### Thermoregulatory Responses

**FIGURE 2:**
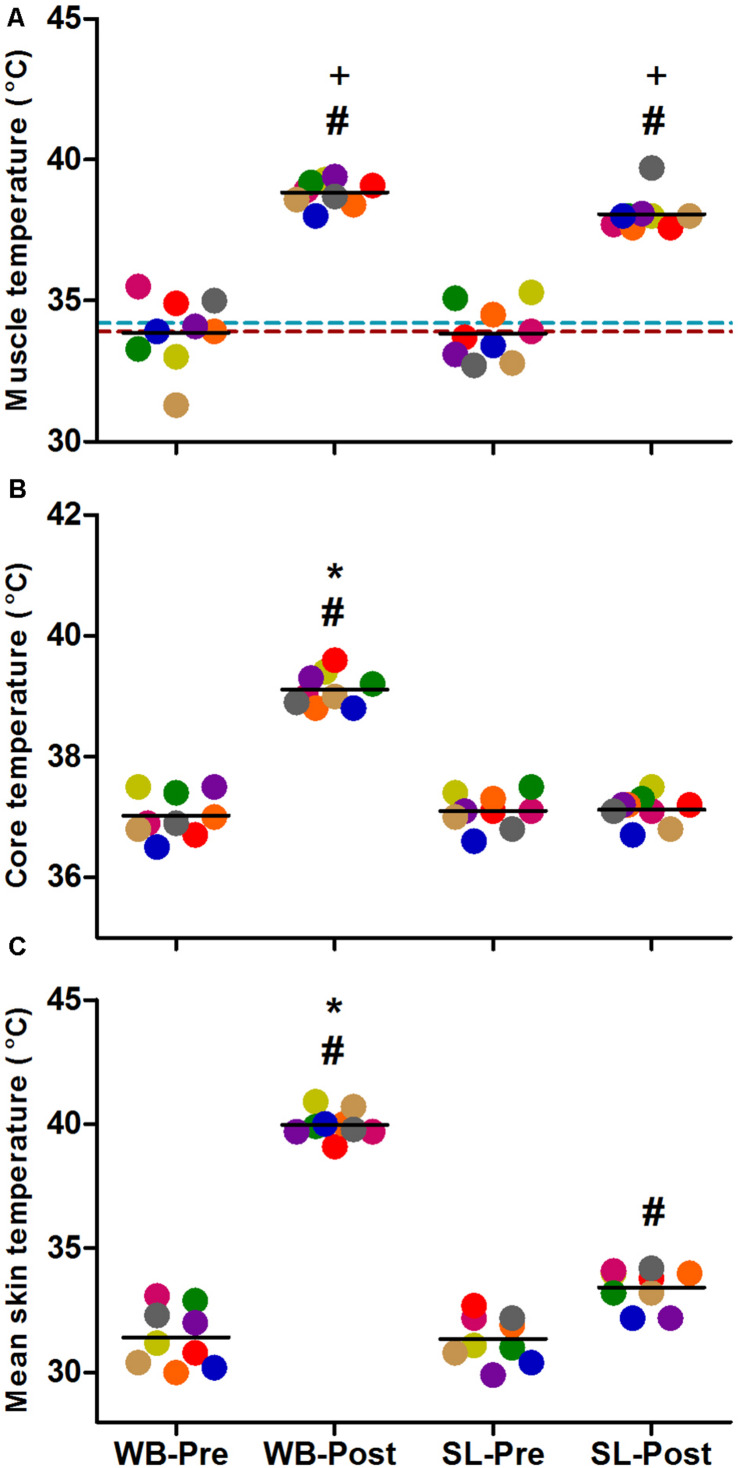
Changes in muscle temperature **(A)**, core temperature **(B)** and mean skin temperature **(C)** during whole body (WB) heat stress and single-leg heat stress (SL). Broken blue and red lines indicate pre and post muscle temperatures, respectively in the CON leg during SL. #different compared with PRE within condition. *Different compared with SL at POST. ^+^Different compared with CON leg during SL at POST.

Changes in T_m_, T_re_, and T_s_ (Figure 2) all demonstrated significant main effects for time, condition and interaction (*p* < 0.0001). Compared with PRE, T_m_ was increased following at POST in WB (38.8 ± 0.5°C, *p* < 0.0001) and SL (38.1 ± 0.6°C, *p* < 0.0001), with no changes observed in CON (*p* = 0.087). At POST, T_m_ in WB and SL were higher compared with CON (*p* < 0.0001), with the difference between WB and SL not reaching statistical significance (*p* = 0.065). T_re_ was significantly higher post WB (39.1 ± 0.3°C) compared with PRE treatment (*p* < 0.0001) or POST SL (*p* < 0.0001). T_s_ was increased in both WB and SL (*p* < 0.0001), with the increase in WB (40.0 ± 0.5°C) greater compared with SL (33.4 ± 0.8°C; *p* < 0.0001) at POST.

### Protein Expression

**FIGURE 3:**
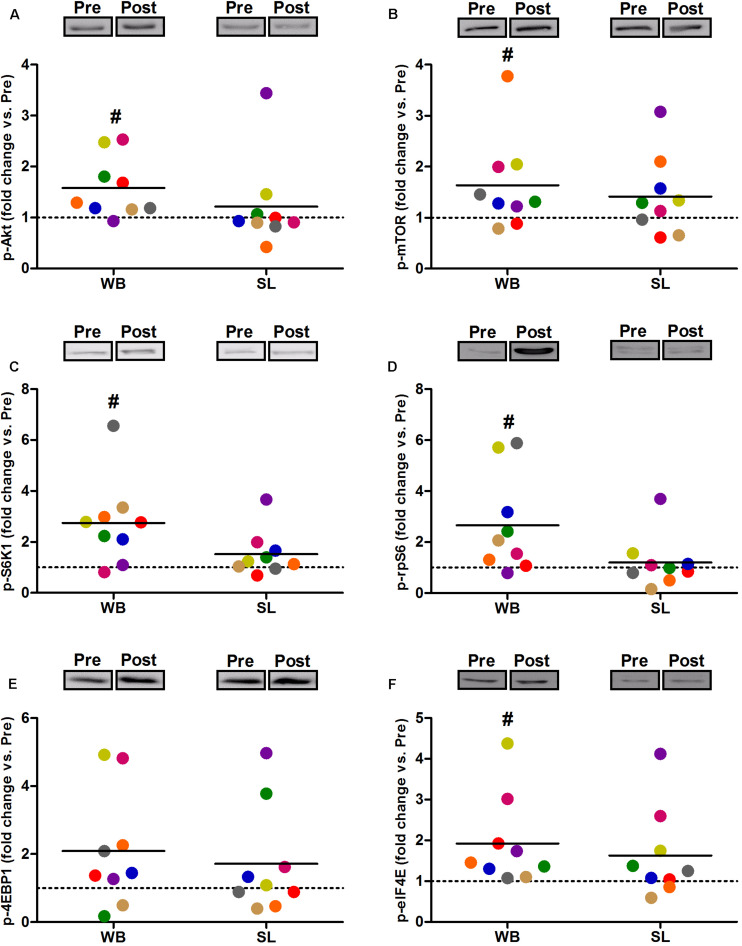
Changes in the phosphorylated state of Akt Ser^473^ **(A)**, mTOR Ser^2448^ **(B)**, S6K1 Thr^389^ **(C)**, rpS6 Ser^235/236^ **(D)**, 4EBP1 Thr^37/46^ **(E)**, and eIF4E Ser^209^ **(F)** during whole body (WB) or single-leg (SL) heat stress. #Different compared with PRE within WB.

**FIGURE 4:**
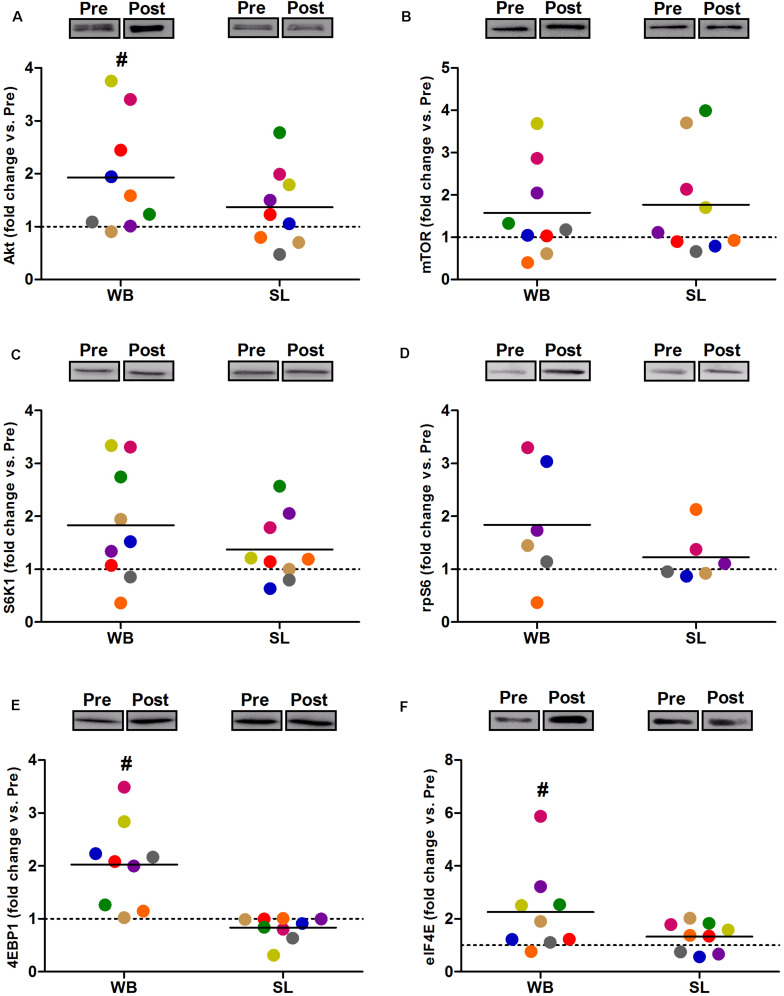
Changes in the total protein expression of Akt **(A)**, mTOR **(B)** S6K1 **(C)**, rpS6 **(D)**, 4EBP1 **(E)**, and eIF4E **(F)** during whole body (WB) or single-leg (SL) heat stress. #Different compared with PRE within WB. *n* = 6 for rpS6 due to technical faults.

Compared with PRE, WB up-regulated p-Akt (58%, *p* = 0.021, ES = 0.52), p-mTOR (64%, *p* = 0.038, ES = 0.49), p-S6K1 (174%, *p* = 0.050, ES = 0.48), p-rpS6 (302%, *p* = 0.038, ES = 0.90), and p-eIF4E (93%, *p* = 0.008, ES = 0.62), with no changes observed in p-4EBP1 (*p* = 0.139, ES = 0.67; Figure 3). Moreover, total protein expressions of Akt (93%, *p* = 0.015, ES = 0.56), 4EBP1 (103%, *p* = 0.008, ES = 0.53), and eIF4E (126%, *p* = 0.010, ES = 0.74) were significantly increased following WB (Figure 4). The phosphorylation status and the total expression of the above proteins were not modified by SL (Figures 3, 4), nor were any changes observed in CON (data not presented; all *p* ≥ 0.070, ES = 0.06–0.54).

**FIGURE 5:**
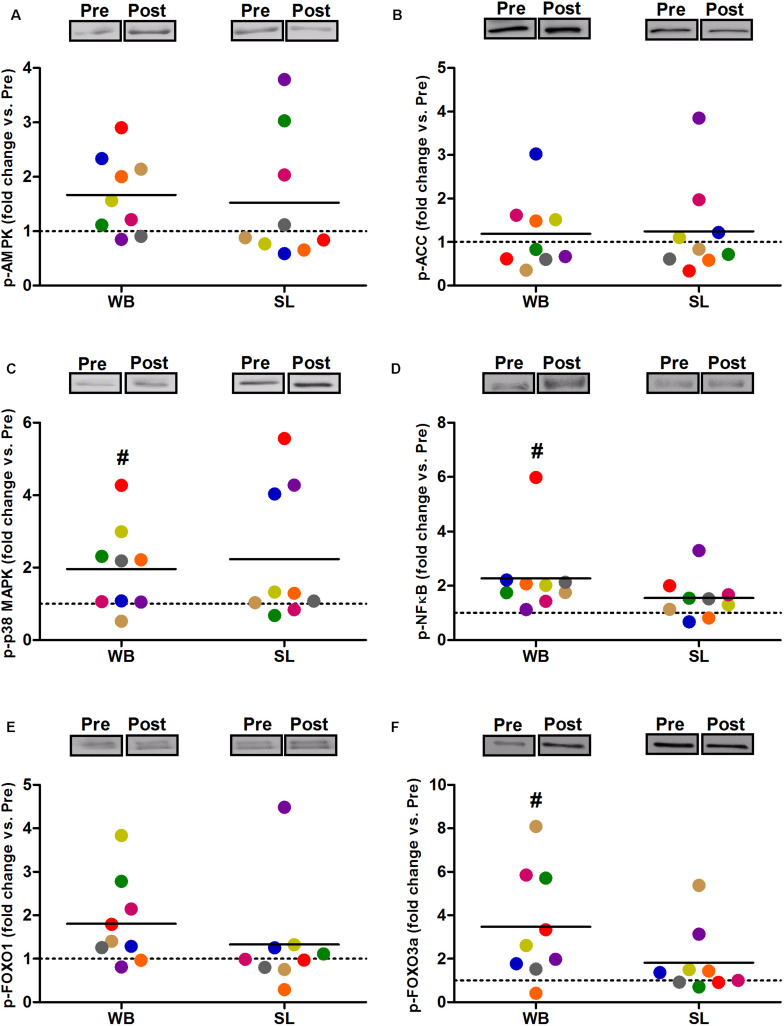
Changes in the phosphorylated state of AMPK Thr^172^ **(A)**, ACC Ser^79^ **(B)** p38 Thr^180^/Tyr^182^ **(C)**, NFκB Ser^536^ **(D)**, FOXO1 Thr^24^ **(E)**, and FOXO3a Thr^32^ **(F)** during whole body (WB) or single-leg (SL) heat stress. #Different compared with PRE within WB.

**FIGURE 6:**
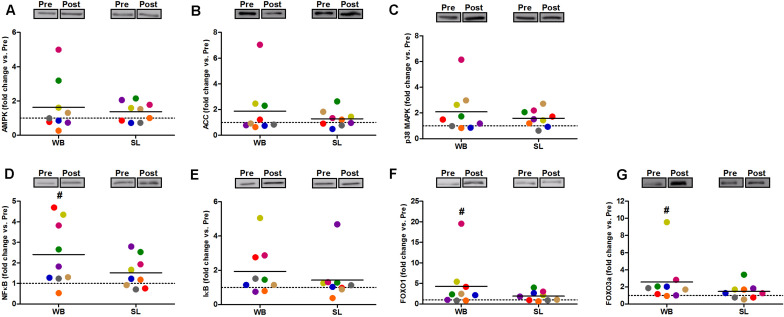
Changes in the total protein expression of AMPK **(A)**, ACC **(B)**, and p38 **(C)**, NFκB **(D)**, IκB **(E)**, FOXO1 **(F)**, and FOXO3a **(G)** during whole body (WB) or single-leg (SL) heat stress. #Different compared with PRE within WB.

In addition, WB increased or tended to increase the phosphorylation status of p38 (97%, *p* = 0.038, ES = 0.34), NFκB (128%, *p* = 0.008, ES = 1.10), FOXO3a (248%, *p* = 0.038, ES = 0.97), AMPK (67%, *p* = 0.051, ES = 0.39), and FOXO1 (81%, *p* = 0.066, ES = 0.74) but not ACC (*p* = 0.953, ES = 0.08; Figure 5). Increases in the total protein expression (Figure 6) following WB were also observed for NFκB (141%, *p* = 0.010, ES = 0.97), FOXO1 (707%, *p* = 0.038, ES = 0.76), and FOXO3a (157%, *p* = 0.015, ES = 0.69), with no changes for AMPK (*p* = 0.767, ES = 0.34), ACC (*p* = 0.767, ES = 0.45), p38 (*p* = 0.139, ES = 0.45), and IκB (*p* = 0.110, ES = 0.54). No changes in phosphorylated or total protein expression were observed following SL (Figures 5, 6) or CON (all *p* ≥ 0.080, ES = 0.01–0.65).

### Gene Expression

**FIGURE 7:**
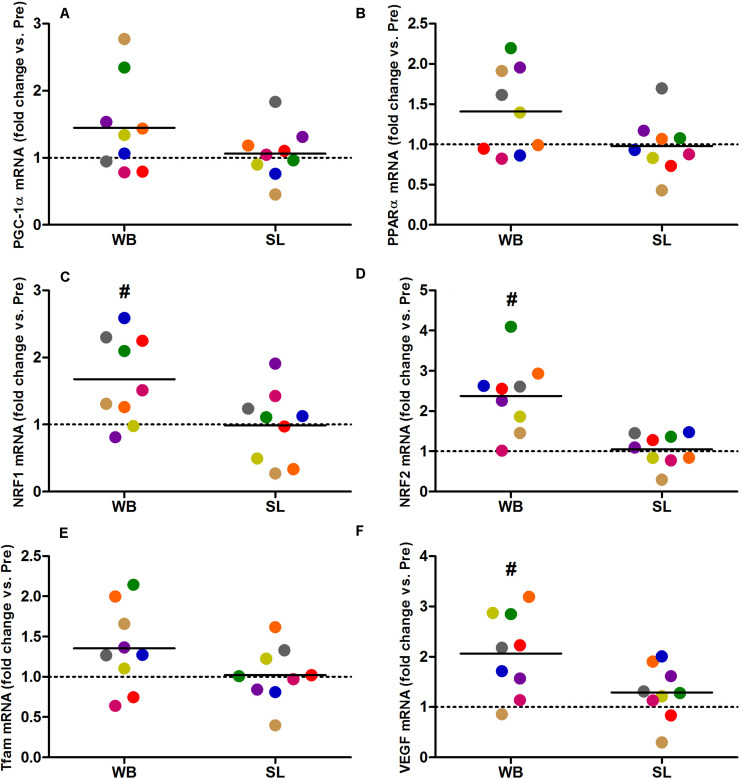
Changes in the mRNA expression of PGC-1α **(A)**, PPARα **(B)**, NRF1 **(C)**, NRF2 **(D)**, Tfam **(E)**, and VEGF **(F)** during whole body (WB) or single-leg (SL) heat stress. #Different compared with PRE within WB.

**FIGURE 8:**
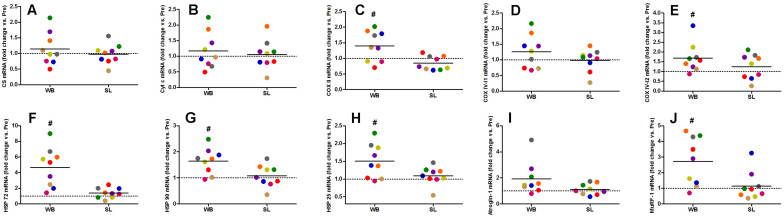
Changes in the mRNA expression of CS **(A)**, Cyt c **(B)**, COX II **(C)**, COX IV-I1 **(D)**, and COX IV-I2 **(E)**, HSP 72 **(F)**, HSP 90 **(G)**, HSP 25 **(H)**, Atrogin-1 **(I)**, and MuRF-1 **(J)** during whole body (WB) or single-leg (SL) heat stress. #Different compared with PRE within WB.

Whole-body or SL demonstrated no influence on the mRNA expression of PGC-1α (WB; *p* = 0.173, ES = 0.67, SL; *p* = 0.859, ES = 0.005) or PPARα (WB; *p* = 0.374, ES = 0.30 SL; *p* = 0.441, ES = 0.08; Figure 7). However, an increase in PGC-1α mRNA was observed following CON (*p* = 0.008, ES = 0.90). Downstream of PGC-1α, WB resulted in increased mRNA expression of NRF1 (68%, *p* = 0.028, ES = 1.01), NRF2 (138%, *p* = 0.008, ES = 1.17), VEGF (107%, *p* = 0.011, ES = 0.86; Figure 7), as well as mitochondrial respiratory subunits COX II (40%, *p* = 0.050, ES = 0.62), and COX IV-I2 (69%, *p* = 0.028, ES = 0.55), but not CS (*p* = 0.953), Cyt c (*p* = 0.767, ES = 0.04), and COX IV-I1 (*p* = 0.441, ES = 0.13; Figure 8). No changes in mRNA expression were observed following SL (Figures 7, 8) or CON (all *p* > 0.090, ES = 0.004–0.54; except in PGC-1α as mentioned above).

Heat shock proteins 72 (362%, *p* = 0.008, ES = 1.39), HSP 90 (64%, *p* = 0.015, ES = 0.96), and HSP 25 (50%, *p* = 0.021, ES = 0.57) were all up-regulated following WB, with no changes observed in SL or CON (all *p* > 0.173, ES = 0.002–0.02; Figure 8). The mRNA expression of Murf-1 was enhanced following WB (173%, *p* = 0.028, ES = 1.00) with no effect observed with SL (*p* = 0.678, ES = 0.0009) or CON (*p* = 0.859, ES = 0.0006). Atrogin-1 tended to increase after WB (92%, *p* = 0.051, ES = 0.69) but was unchanged following SL (*p* = 0.767, ES = 0.20) or CON (*p* = 0.953, ES = 0.05; Figure 8).

## Discussion

The present study aimed to profile the molecular responses and pathways in response to WB and localized heat stress in human skeletal muscle. The main findings are that WB: (1) enhanced Akt/mTOR signaling; (2) increased phosphorylation and thereby inactivation of FOXO3a; (3) up-regulated the mRNA levels of HSP 72, 90, and 25, and (4) increased the gene expression of several downstream targets despite no change in PGC-1α mRNA levels. Given that WB upregulated Akt/mTOR signaling and HSP mRNA levels as well as induced an increase in several genes related to mitochondrial biogenesis, WB heat treatment might beneficially influence the regulation of skeletal muscle mass in humans. However, as WB also tended to up-regulate the mRNA levels of proteolytic atrogenes, further investigation is warranted. Conversely, our data do not show any significant effect of SL on any of the investigated parameters.

### Molecular Responses to WB

Our findings here corroborate with previous studies in rodents (Uehara et al., 2004; Ohno et al., 2012; Yoshihara et al., 2013) and cell cultures (Obi et al., 2019) showing an activation of the Akt/mTOR pathway following heat exposure. However, the mechanism(s) by which heat stress may upregulate Akt/mTOR, and consequently downstream signaling are not well understood. Potential candidates are HSPs, as suppressed phosphorylation of Akt and S6K1 were observed following heat stress in heat shock factor one gene deficient mice (Ohno et al., 2015). Moreover, treatment with a HSP 72 inducer was shown to upregulate Akt and S6K1 phosphorylation in both control and HSP 72 knockdown L6 myotubes (Gwag et al., 2015). More recently, treatment with HSP 70 antagonists was shown to abolish heat-induced phosphorylation of Akt in differentiating C2C12 mouse myoblast (Obi et al., 2019). These findings indicate that the Akt/mTOR pathway may be upregulated through a HSP-mediated mechanism following heat stress. Although we did not assess the protein expression, the current results show an increase in HSP 72, 90, and 25 mRNA levels, supporting a HSP-centered mechanism to account for the increased phosphorylation of the Akt/mTOR pathway. Nevertheless, other mechanisms independent of HSP may also regulate the Akt/mTOR pathway following heat stress. For instance, an increase in Akt and S6K1 phosphorylation has been reported in rodents subjected to varying levels of heat stress (30 min at 39–41°C), despite no increase in HSP 72, 90, and Heat Shock Cognate protein 73 expression (Yoshihara et al., 2013). Thus, it is likely that a combination of HSP-dependent and HSP-independent mechanisms contributed to the increase in Akt/mTOR signaling observed in the present study.

Consistent with the increase in HSPs, an increase in the mRNA levels of VEGF, NRF1, NRF2, as well as several respiratory chain complexes (i.e., COX II and COX IV-I2) were observed following WB. Our findings support the vascular benefits of WB heat treatment, in agreement with recent work in healthy humans demonstrating increased angiogenic regulators (including VEGF mRNA) following 90 min of lower body passive heat therapy (Kuhlenhoelter et al., 2016), with longer term WB heat therapy for 6 weeks (40–50 min per session, 3× per week) resulting in enhanced skeletal muscle capillarization (Hesketh et al., 2019). However, our findings differ from work reporting repressed expression of PGC-1α mRNA expression, or no change in mitochondrial density following acute (Petrie et al., 2016) and longer term heat therapy, respectively (Hesketh et al., 2019). Instead, our findings in general corroborate with data acquired in cell and rodent models showing an increase in markers associated with mitochondrial biogenesis (Liu and Brooks, 2012; Tamura and Hatta, 2017), which have been extended to human models demonstrating a HSP-associated increase mitochondrial adaptation in healthy weight-bearing as well as immobilized humans (Hafen et al., 2018, 2019). We did not observe increased PGC-1α mRNA levels in the current study, despite the increase in gene transcription coding for mitochondria-related proteins. Due to technical challenges (i.e., unavailability of commercially available antibodies suitable for human muscle tissue), we did not assess the nuclear protein expression nor the transcriptional activity, but as PGC-1α is a key upstream regulator and coordinator of the mitochondrial biogenesis program (Puigserver et al., 1998; Wu et al., 1999), we may not rule out that it contributed to the increased expression of mitochondria-related genes here. Besides a HSP-mediated mechanism regulating mitochondrial-related genes, we looked at the phosphorylation state of p38 MAPK and AMPK, key kinases known to activate PGC-1α (Akimoto et al., 2005; Jager et al., 2007). In heat stress models, an increase in AMPK activity has been purported to account for PGC-1α activation and mitochondrial biogenesis *in vitro* (Liu and Brooks, 2012) but not in rodents *in vivo* (Tamura et al., 2014). In the latter model, p38 seems to be the principal upstream regulator of PGC-1α (Tamura et al., 2014). In line with this previous study (Tamura et al., 2014), an increase in p38 phosphorylation was found following WB, which may have contributed to the increase in mitochondrial-related responses observed in the present study.

The present study provides novel data regarding the regulation of FOXO transcription factors following heat stress in human skeletal muscle. The increased FOXO3a and tendency toward increased FOXO1 phosphorylation could be due, at least partially to the increase in Akt phosphorylation, as the latter has been shown to phosphorylate FOXO (Sandri et al., 2004). The phosphorylation of FOXO transcription factors constitutes an important step in limiting atrophic processes, as it retains this factor(s) within the cytosol and inhibits translocation into the nucleus where it upregulates the transcription of proteolytic genes (Brunet et al., 1999). Key atrogenes upregulated by FOXO include Murf-1 and Atrogin-1 (Bodine and Baehr, 2014), and purportedly account for 80% of proteolysis during muscle atrophy (Tawa et al., 1997). In line with increased phosphorylation of FOXO3a and FOXO1 following WB, a downregulation in Murf-1 and Atrogin-1 mRNA was expected. In contrast, Atrogin-1 mRNA tended to increase, whilst a significant increase in Murf-1 mRNA was observed following WB, indicating that transcription of Murf-1 and Atrogin-1 mRNA levels in the present study may have been regulated by A FOXO independent mechanism. While the role of FOXO in regulating the transcription of Murf-1 and Atrogin-1 in response to immobilization or fasting has been evidenced repeatedly, there is considerable uncertainty whether any of the FOXO family members regulate the expression of these atrogenes in the absence of catabolic stimuli (Senf et al., 2009). Alternatively, our findings report increased phosphorylation of NFκB following WB. Increased gene expression of Murf-1 and Atrogin-1 have been associated with increased NFκB activity in disuse models (Bodine and Baehr, 2014; Egerman and Glass, 2014). However, further work is required to verify NFκB as a potential upstream regulator of these atrogenes following WB heat stress.

### Molecular Responses to SL

In humans, regulation of HSPs have been primarily investigated following localized heat stress models, with considerable differences in findings (Morton et al., 2007; Hafen et al., 2018, 2019). For instance, despite substantial increases in core (∼1.5°C) and muscle (∼3.6°C) temperatures, 60 min of one-legged hot water immersion has been shown to demonstrate no effect on the mRNA levels of HSP 27, HSP 60, or HSP 70 (Morton et al., 2007). Moreover, protein expressions of HSP 70 and HSP 90 were not altered following 8 weeks of localized heat therapy to the quadriceps (Kim et al., 2020). In contrast, a recent study reported increased HSP mRNA levels following 90 min of localized quadriceps heat therapy (Kuhlenhoelter et al., 2016). An increase in HSP 70 and HSP 90 protein expressions have been observed in weight-bearing and immobilized participants following quadriceps muscle heat treatment administered for 120 min for 6–10 consecutive days (Hafen et al., 2018, 2019). As we did not find any changes in the mRNA content of any of the HSPs following SL, we could speculate that a single episode of localized heating may be insufficient to upregulate the HSP response, unless the duration and intensity of the heat treatment is adequately potent. Indeed, our treatment time was limited to 60 min, which is substantially shorter compared with the aforementioned studies (90–120 min; Kuhlenhoelter et al., 2016; Hafen et al., 2018, 2019). Moreover, the muscle temperatures attained following SL (38.1 ± 0.6°C) in our study were considerably lower compared with those reported previously [∼40°C (Hafen et al., 2018) and 39.5°C (Morton et al., 2007)]. Nevertheless, for the same duration (i.e., 60 min), the current study indicates that WB treatment, resulting in substantial increases in core, skin and muscle temperatures may be a more potent stimulus to activate the HSP response compared with localized treatment.

In line with our HSP data, SL did not up-regulate the Akt/mTOR pathway, again likely owing to the lower peak muscle temperatures achieved following SL. In support, a temperature-dependent phosphorylation of Akt and S6K1 was found in rodents exposed to varying degrees of heat stress (37–41°C; Yoshihara et al., 2013). Significant increases in Akt and S6K1 phosphorylation were observed at 39°C and onward, with the most pronounced activation measured at 41°C. Here, a muscle temperature of 38.1°C following SL was perhaps insufficient to induce appreciable changes as compared to the 38.8°C reached in WB. Nevertheless, it cannot be ruled out that the concomitant increase in core and overall body temperatures could have also contributed toward the enhanced Akt/mTOR signaling response seen by others in rodents (Yoshihara et al., 2013) and here in WB.

In contrast to recent studies (Hafen et al., 2018, 2019), no effect of SL was observed on any of the parameters related to mitochondrial adaptations. Consistently, we found no effect of SL on upstream kinases (i.e., AMPK, p38 MAPK) regulating the mitochondrial biogenesis program. Instead, our findings align with Kim et al. (2020), who demonstrated no effect on mitochondrial adaptations following 8 weeks of localized (quadriceps) heat therapy. As discussed, such divergent findings may be attributed to differences in the duration of heating and changes in muscle temperature. In addition, heating modality may also account for the observed differences. Specifically, pulsed shortwave diathermy utilized by Hafen et al. (2018, 2019) enables rapid and substantial deep tissue heating, and resultantly may be more effective at activating mitochondrial adaptations compared with superficial heat modalities as employed in the current and previous report (Kim et al., 2020). Taken together, the current and previous findings (Hafen et al., 2018, 2019; Kim et al., 2020) tend to indicate muscle temperature, core temperature and duration of stimulus as important factors that determine the extent to which whole body or localized heat stress may be beneficial.

In summary, the present study reports that WB heat stress enhanced anabolic signaling (Akt/mTOR), upregulated HSPs mRNA levels, enhanced the gene expression of several targets associated with mitochondrial biogenesis, and inhibited the activity of FOXO transcription factors, although increased expression of Murf-1 was observed. Conversely, 60 min of localized heat treatment resulting in peak muscle temperatures of ∼38.1°C demonstrated no influence on the investigated signaling pathways or mRNA levels. While further research is needed to optimize heat therapy protocols and dose-response, the present work demonstrates that WB heat stress may beneficially influence the regulation of skeletal muscle mass in humans, highlighting the potential use of this modality in both sport and clinical situations.

## Data Availability Statement

The raw data supporting the conclusions of this article will be made available by the authors, without undue reservation, to any qualified researcher.

## Ethics Statement

The studies involving human participants were reviewed and approved by Anti-Doping Laboratory Research Ethics Committee. The patients/participants provided their written informed consent to participate in this study.

## Author Contributions

MI, LD, and SR conceived and designed the research. MI, JM, AC, and SR conducted the experiments. MI, AC, FB, LD, and SR performed the data analysis. MI, FB, LD, AC, JM, and SR assisted with the data interpretation. MI, LD, and SR wrote the manuscript. All authors read and approved the manuscript.

## Conflict of Interest

The authors declare that the research was conducted in the absence of any commercial or financial relationships that could be construed as a potential conflict of interest.
