# Supplementary material for: Skeletal Muscle Signaling Following Whole-Body and Localized Heat Exposure in Humans
Source: Front Physiol. 2020 Jul 14;11:839. doi: 10.3389/fphys.2020.00839 (PMC7381176; doi:10.3389/fphys.2020.00839)
Supplement: Supplementary file 1 [file Presentation_1.pptx]

## Slide 1
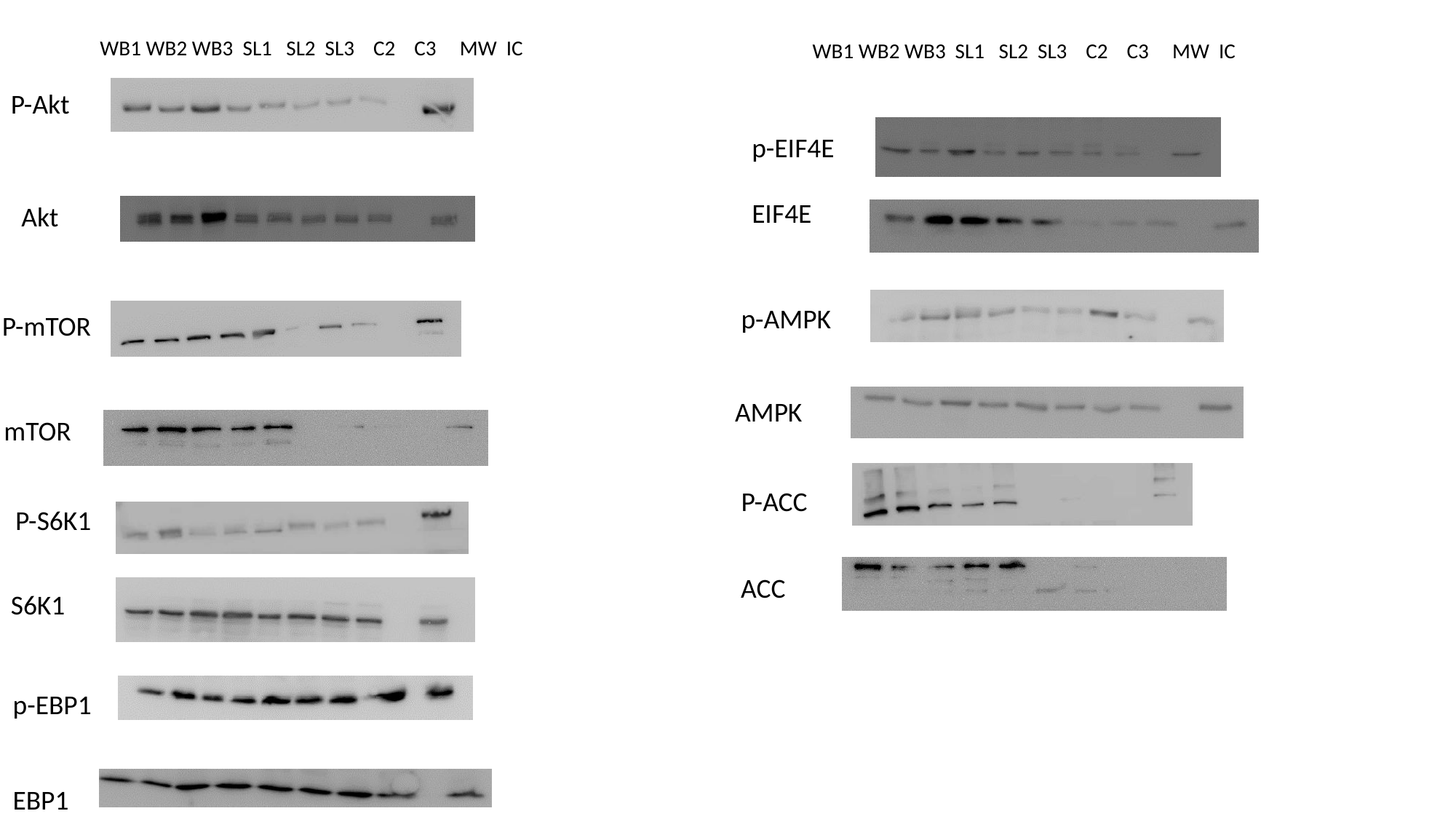

WB1 WB2 WB3 SL1 SL2 SL3 C2 C3 MW IC
 WB1 WB2 WB3 SL1 SL2 SL3 C2 C3 MW IC
P-Akt
p-EIF4E
EIF4E
Akt
p-AMPK
P-mTOR
AMPK
mTOR
P-ACC
P-S6K1
ACC
S6K1
p-EBP1
EBP1

## Slide 2
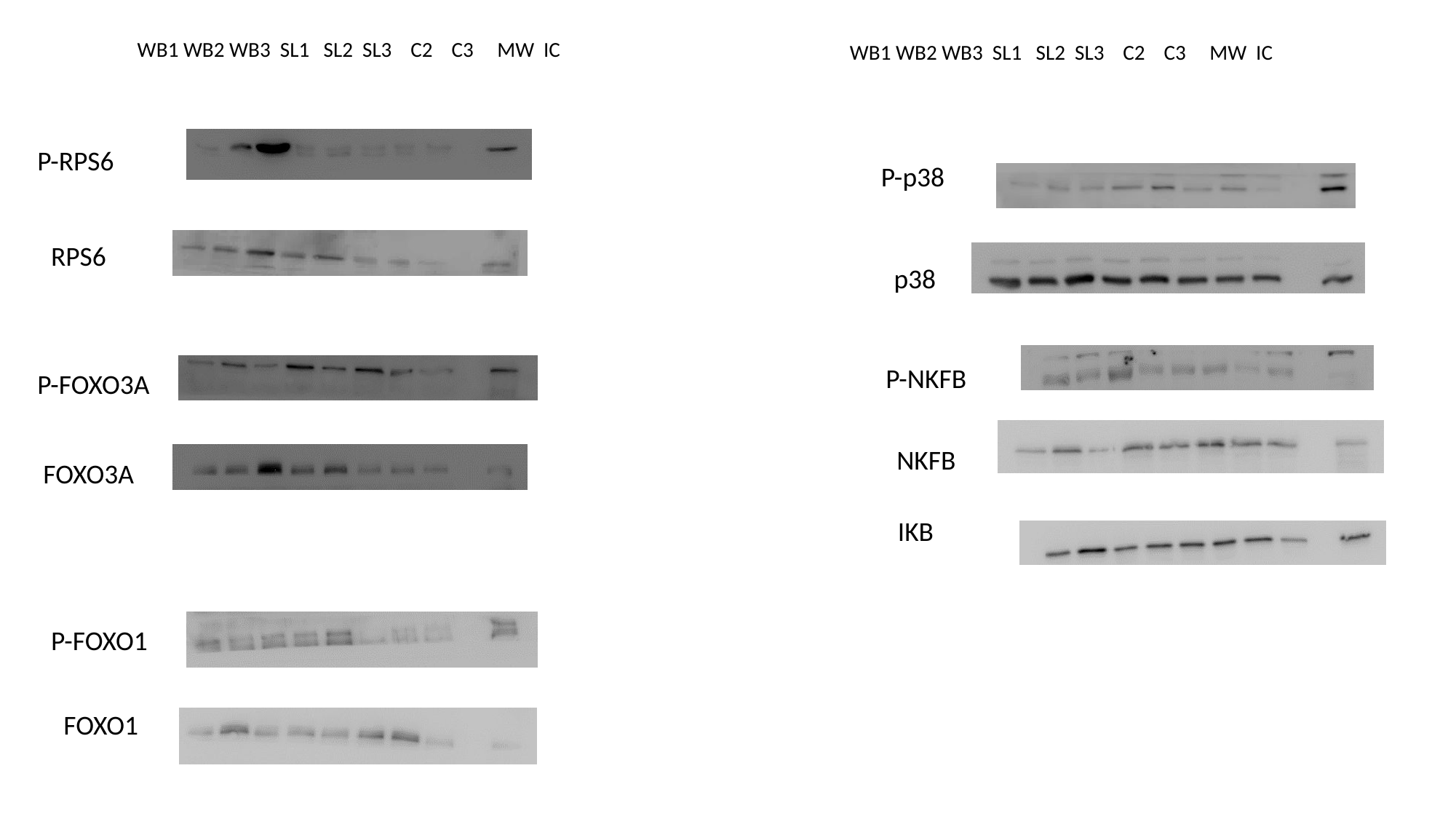

WB1 WB2 WB3 SL1 SL2 SL3 C2 C3 MW IC
 WB1 WB2 WB3 SL1 SL2 SL3 C2 C3 MW IC
P-RPS6
P-p38
RPS6
p38
P-NKFB
P-FOXO3A
NKFB
FOXO3A
IKB
P-FOXO1
FOXO1
